# In Vitro Sprouted Plantlets of *Citrullus colocynthis* (L.) Schrad Shown to Possess Interesting Levels of Cucurbitacins and Other Bioactives against Pathogenic Fungi

**DOI:** 10.3390/plants11202711

**Published:** 2022-10-14

**Authors:** Belsem Marzouk, Meher Refifà, Serena Montalbano, Annamaria Buschini, Stefano Negri, Mauro Commisso, Francesca Degola

**Affiliations:** 1 Laboratory of Chemical, Galenic and Pharmacological Development of Drugs, Faculty of Pharmacy, University of Monastir, Monastir 500, Tunisia; 2 Department of Chemistry, Life Science and Environmental Sustainability, University of Parma, 43124 Parma, Italy; 3 Interdepartmental Centre for Molecular and Translational Oncology COMT, University of Parma, 43124 Parma, Italy; 4 Department of Biotechnology, University of Verona, 37134 Verona, Italy

**Keywords:** cucurbitacins, *Citrullus colocynthis* (L.) Schrad, in vitro sprouted plantlets, anti-mycotoxigenic activity, antifungal compounds, *Candida* spp., *Aspergillus flavus*

## Abstract

Cucurbitacins, structurally different triterpenes mainly found in the members of Cucurbitaceae, possess a vast pharmacological potential. Genus *Cucurbita*, *Cucumis,* and *Citrullus* are affluent in these bioactive compounds, and, amongst them, *Citrullus colocynthis* (L.) Schrad. is widely exploited in folk medicine, since a huge number of diseases are successfully treated with organic and aqueous extracts obtained from different organs and tissues of the plant. The well-known pharmacological activities of such species have been attributed to its peculiar composition, which includes cucurbitacins and other bioactive molecules; thus, owing to its high importance as a valuable natural resource for pharmaceuticals and nutraceuticals, *C. colocynthis* propagation and multiplication protocols are considered significant, but the exploitation of its phytochemical potential is limited by the restricted cultivation conditions and the low rate of seed germination in the natural environment; in fact, the assessment of accumulation rate of specific phytochemicals under controlled conditions is still missing. Axenically sprouted plantlets obtained without the use of culture media or the addition of hormones have been evaluated here for the production of bioactive compounds and relevant bioactive features. Our results proved that derived organic extracts contain cucurbitacins and other bioactives, show antioxidant potential, and exert activity against some pathogenic fungi (*Candida krusei, C. albicans, C. parapsilosis, C. glabrata*, and *Aspergillus flavus*), supporting the feasibility of a methodology intended to scale-up cultivation of this species as a source of pharmaceutically interesting compounds, achievable from plantlets cultivated under laboratory conditions.

## 1. Introduction

According to the World Health Organization (WHO), more than 80% of the population of developing countries still depend on traditional medicine for the treatment of most common diseases [1] (accessed 5 May 2022). Many prescriptions from traditional practitioners are now available in pharmacies, supermarkets, and local markets, and refer to plant materials mainly exported by India, China, Africa, and Central America. At the same time, the diffusion of non-medical, holistic approaches—such as phyto- and aromatherapy—represents a strong business driver for the exploitation of the most variety of plant species for the mining of new principles. While some of these drugs currently come from cultivated plants, the majority is still obtained from savage species; accordingly, the last decades have been characterized by a rising interest in the individuation of pharmacologically active extracts and/or purified compounds from an ever wider number of plant species, along with the definition of their mechanism of action. Despite the fact that the cultivation of medicinal plants offers an excellent resource for constant availability and overexploitation, users still perceive cultivated plants as less effective in comparison to wild ones, as a result of their domestication process [2,3]. In fact, due to the observation that the majority of bioactive compounds are synthesized by plants as both constitutive and inducible defense during exposure to stress, as well as secondary metabolites—and mainly those with antimicrobial activity—are produced in response to pathogen attack [4,5], it is a truth universally acknowledged that domestication of wild plants led them to fail in synthesizing most of the biologically active molecules, as a result of natural selection exerted by the maintenance in a controlled agricultural ecosystem in which organisms are less prone to environmental stimuli alterations. Hence, cultivated individuals seem to be usually less resistant to biotic stresses compared to their wild counterparts because less abundant in defense compounds, that are known to be triggered by either biotic or abiotic elicitors [6]. When it comes to the advantages of in vitro culturing plants in comparison with conventional greenhouse or in-field techniques, the possibility of controlling developmental processes (such as organogenesis), to obtain homogenous material, and producing a larger amount of tissues/organs interested by desirable metabolites content, are the most appreciated; but, in these controlled conditions, the synthesis of most of the secondary metabolites—and bioactives, in particular—should be to greater reason even lower than in a natural environment. To date, the lack of scientific evidence on the potency of cultivated medicinal plants remains a limiting factor for the development of cultivation techniques for pharmacologically interesting but recalcitrant wild species; there is therefore a need to validate this claim, exploring the real possibilities of obtaining still “chemically rewarding” cultivated (or in vitro propagated) plants on a case by case basis.

*Citrullus colocynthis* (L.) Schrad., one of the most fascinating plants traditionally used in African and Asian folk medicine with various ethnopharmacological purposes, is a well-known cucurbit with reported uses against a huge number of diseases: from dermatological, gynecological, and pulmonary infections, to inflammatory, cardiovascular, and immune-related disorders [7]. Several are the bioactive compounds that, according to the literature, have been found in different *C. colocynthis* organs [8,9]: mainly attributed to glycosides, flavonoids, alkaloids, and essential oil families; some of them have been demonstrated to possess interesting antimicrobial properties against pathogenic fungal species [10,11,12,13]. This plant is therefore regarded as a promising source for bioactives, to be used in both biocontrol strategies for crop protection and investigated for the design of new generation drugs [14,15,16]; despite the number of studies exploring the biological activities of crude and/or organic extracts from the most various organs of *C. colocynthis*, only a few reports describe the isolation and characterization of single chemical constituents. Detailed phytochemical characterizations of extracts from wild plants confirmed the presence of different classes of metabolites (coumarins, hydroxycinnamic acid derivatives, flavan-3-ols glycosides, flavone glycosides, and tetracyclic triterpenes) [17,18,19], that have been indicated as responsible for antifungal activity against *Aspergillus* strains [20]; more recently, esculetin, p-coumaric acid derivatives, orientin, vitexin, apigenin derivatives, epicatechingallate, and Cucurbitacins (E and I) have been reported as the major components characterizing chloroform and methanol leaves, stem, and root extracts that possessed an anti-aflatoxigenic effect on the phytopathogenic fungus *Aspergillus flavus* [12]. Due to the implications that these findings could have on the future development of new drugs—and their commercial use—any effort to fine-tune the possible propagation and cultivation methodologies for this particular plant species is desirable, as long as the maintenance of the relevant phytochemical features is assured. Hence, the determination/validation of bioactive content and biological activity of cultivated material is mandatory. In this sense, Cucurbitacins accumulation in callus cultures was investigated in *Trichosanthes cucumerina* and *Citrullus colocynthis* [21,22]: results showed that under hormones-mediated stimulation, a certain production level can be obtained, suggesting that an in vitro strategy for the achievement of these secondary metabolites could be pursued. However, in vitro cultured cells are prone to somaclonal variations due to genetic instability, often resulting in the loss of characteristic traits, for instance, the production of certain secondary metabolites [23]. To our far knowledge, this is the first phytochemical characterization of bioactive extracts obtained from in vitro sprouted and hydroponically grown-*C. colocynthis* plantlets. The biological activity of plantlet extracts on *A. flavus* was also compared to the effect of the seed coat (tegument) extract, in the forward-looking perspective of a circular economy.

## 2. Materials and Methods

### 2.1. Plant Material

#### 2.1.1. *C. colocynthis* Seeds Provenience

Plant seeds were collected in the municipality of Sidi Makhlouf (Medenine, Tunisia). The identification was conducted on the relevant adult plants and was conducted according to a voucher specimen (C.C-01.01), deposited in the Faculty of Pharmacy of Monastir University (Tunisia), and following the “Flora of Tunisia” [24].

#### 2.1.2. Seeds Germination and Plantlets Achievement

In order to obtain the highest ratio of germination, seeds were firstly screened for their viability by dipping in a water-filled glass jar: those that floated on the water surface were discarded as less viable. Selected seeds (about 50) were manually decoated using a surgical blade, paying high attention to avoid the removal/injury of the embryo, and were then surface sterilized for 10 min in a diluted bleach solution (0.1% *v*/*v* Tween20; final concentration 5% *v*/*v* of a sodium hypochlorite commercial stock). The seeds’ tegument was conserved for further phytochemical analyses. After sterilization, seeds were washed five times with sterile bi-distilled water to completely remove the sodium hypochlorite, were laid between two layers of autoclaved cotton wool into sterilized glass jars, and added with 6 mL of bi-distilled sterile water. Jars were incubated in the dark at 28 °C until seed germination, approximately 5–6 days; after germination, the upper layer of cotton wool was axenically removed, and jars were transferred in a plant growth chamber at 25 °C with a 16/8 h light/dark photoperiod (150 µmol photons m^−2^ s^−1^). After four days of incubation, cultures were added with 3 mL of a nutrient sterile solution (1/4 strength Murashige-Skoog liquid medium [MS salts; Duchefa; #M0222, Haarlem, The Netherlands]; 2% *w*/*v* sucrose), and maintained in the same growth conditions until the development of the first two true leaves (about 1 week); then, plantlets were transferred in plastic, opaque, open-top boxes, and maintained in hydroponic cultivation (1/4 strength Murashige-Skoog liquid medium, without root oxygenation; 25 °C; 16/8 h light/dark photoperiod, 150 µmol photons m^−2^ s^−1^ ). At the stage of 6–7 leaves, the plantlets were collected, separated from roots, lyophilized, and processed for analyses.

### 2.2. Preparation of Crude Extracts

The extraction was performed on 7 g of lyophilized plant material and 2 g of seed coats ground to powder: samples were placed inside a thimble made of thick filter paper, and loaded into the main chamber of the Soxhlet extractor. Chloroform and ethanol solvents, in ascending polarity, were used for Soxhlet extraction to fractionate the soluble compounds from the plant material. The total extracting time was 6 h for each solvent continuously refluxing over the sample. The resulting extracts were evaporated at reduced pressure to obtain the crude extracts, which were then dissolved in dimethyl sulfoxide (DMSO).

### 2.3. Crude Extracts Characterization

#### 2.3.1. Determination of Total Phenolics Content

The concentration of total phenolic compounds was determined in the different extracts according to Singleton and Rossi [25], by using the Folin–Ciocalteu’s phenol reagent. Briefly: 100 µL of the sample was added to 100 µL of Folin–Ciocalteu’s reagent; after 3 min, 100 µL of a saturated sodium carbonate solution was added to the mixture and adjusted to 1 mL with bi-distilled water. The reaction was kept in the dark for 90 min, then absorbance was measured at 720 nm. The total phenolic content was calculated as mg of gallic acid equivalents/g of crude extract.

#### 2.3.2. Phytochemical Characterization

LC-MS sample preparation: Extract samples corresponding to 45 mg of dry weight and dissolved in DMSO were dried with a speed-vac system (Heto-Holten; Frederiksborg, Denmark). The resulting pellet was dissolved in 450 µL of methanol LC-MS grade (Honeywell, Seelze, Germany). Samples were sonicated for 10 min in an ultrasonic bath (40 kHz; SOLTEC, Milano, Italy) in ice, centrifuged for 10 min at 14,000× *g* at 4 °C and the supernatant diluted 1:50 (*v*/*v*) with water LC-MS grade (Honeywell, Seelze, Germany). Subsequently, samples were centrifuged for 10 min at 14,000× *g* at 4 °C and the supernatants were collected and filtered with 0.22 μm Minisart filters (Sartorius Stedim Biotech, Germany). Samples of in vitro cultivated seedlings previously extracted with ethanol and chloroform were directly subjected to LC-MS analysis and 0.5 µL were injected. Samples of leaves previously extracted in chloroform and seeds with ethanol were further diluted 1:10 and 1:2 (*v*/*v*) with water LC-MS grade (Honeywell, Seelze, Germany), respectively. Then, 0.1 and 0.2 µL were injected for leaf and seed extracts.

LC-MS conditions and analysis: The LC-MS device consisted of an ACQUITY I CLASS UPLC system (Waters, Milford, MA, USA) online with a lambda PDA detector and a Xevo G2-XS qTOF mass spectrometer (Waters) mounting an ESI ion source operating in either positive or negative mode. Molecules were separated by using a Waters ACQUITY UPLC BEH C18 column (2.1 mm × 100 mm, 1.7 μm) with a BEH C18 guard column (2.1 mm × 5 mm, 1.7 μm) (Waters). The chromatographic method lasted 25 min and employed water acidified with 0.1% formic acid as solvent A and 100% acetonitrile as solvent B. The gradient started and stayed at 1% B for 1 min, then increased to 40% B in 9 min, to 70% B in 3.5 min, to 90% B in 1.5 min, to 100% B in 1.5 min, remained at 100% B for 4.5 min and then decreased to 1% B in 0.1 min and kept this isocratic condition for 4.9 min (re-equilibrium). The flow rate was set to 0.350 mL/min and the column was kept at 30 °C. The autosampler SM-FTN (Sample Manager with Flow-Through Needle) was kept at 8 °C. MassLynx v4.1 (Waters) and was used to monitor and control all the instrument functions.

The mass spectrometer parameters, including the ion source and qTOF values, were those previously reported [26].

Raw Data Processing and Identification of the Metabolites: The LC-MS raw data were processed through Progenesis QI software (Waters) by using default parameters. The identification process was initially performed by using the *m*/*z* ratios, isotope similarities, and fragmentation patterns of the detected metabolites and the automatic online search tool available in Progenesis QI for the research in public databases (MassBank, PlantCyc, Plant Metabolic Network, and Human Metabolome Database). Moreover, all the identifications were manually checked by using data reported in the literature as well [7,19,27,28].

### 2.4. Microorganisms and Biological Assays

#### 2.4.1. Fungal Strains

*Candida* spp. strains were obtained from the ATCC collection: *Candida albicans* ATCC 90028, *Candida glabrata* ATCC 90030, *Candida krusei* ATCC 6258, and *Candida parapsilosis* ATCC 22019 were used in this study. The *Aspergillus flavus* non-toxigenic strain TOφ and the aflatoxigenic strain CR10 were used [29] and are available from the authors upon request.

#### 2.4.2. Agar Well Diffusion Assay for Antifungal Activity Evaluation on *Candida* spp.

Extracts were screened for antimicrobial activity using an agar diffusion technique [30]: cells from *Candida* spp. cultures were collected with a sterile loop and dissolved in 10 mL Mueller-Hinton broth, adjusted to obtain a suspension of 10^9^ cells/mL, then seeded on blood Mueller-Hinton Petri dishes poured with 20 mL medium; plates were incubated for 30 min at 37 °C to allow a complete fixation of cells in the overlaid agar. Wells of 6 mm diameter were obtained in the agar medium with a sterile cork-borer and were then filled with 15 µL of 10% *v*/*v* DMSO dissolved crude extracts. Plates were then left undisturbed until the complete diffusion of extract into the agar, and incubated in the dark at 37 °C. Zones of growth inhibition were measured after 48 h of incubation. Positive control was represented by plates amended with Amphotericin B 100 µg/mL.

#### 2.4.3. Radial Growth Assay for Antifungal Activity Evaluation on *A. flavus* Aflatoxigenic Strain

Conidia suspensions were obtained from 10-days old *A. flavus* cultures on YES-agar medium [2% (*w*/*v*) yeast extract (Difco, Detroit, MI, USA); 5% (*w*/*v*) sucrose (Sigma, St Louis, MO, USA); 2% (*w*/*v*) agar (Difco)]: according to Degola et al., 2012 [31], YES-agar medium sections inoculated with *A. flavus* strains were placed axenically into 10 mL glass tubes with 500 μL of sterile bi-distilled water, incubated at 28 °C until sporification was complete, then washed with 2 mL of sterile washing solution (bi-distilled water supplemented with 0.01% Tween20). The suspension was filtered to remove mycelium fragments and other CFUs, then conidia concentration was quantified spectrophotometrically and adjusted by appropriate dilutions to 10^5^ spores mL^−1^. Determination of *A. flavus* radial growth was performed in Petri dishes poured with 18 mL of YES-agar medium amended with 500 μg/mL extracts (or an equal volume of DMSO as control) and spotted on the surface with 5 μL of spore suspension in three equidistant points.

Plates were sealed with Parafilm^®^ and incubated at 25 °C in the dark for up to 5 days; colonies’ radii (mm) were measured at 2, 3, 4, and 5 days post-inoculation. Two measures were recorded daily for each colony, for a total of 6 measures/plate/day, and 3 independent plates were replicated for each treatment.

#### 2.4.4. Multiwell Assay for Anti-Aflatoxigenic Activity Evaluation

A high-throughput procedure performed in multiwell plates was used to assess aflatoxin accumulation in a coconut-milk-derived medium (CCM), by a fluorescence-based method previously described [12,31]. Conidial suspensions from TOφ or CR10+ strain, obtained as reported above, were diluted to the final concentration of 5 × 10^2^ conidia/well and inoculated in a 96-wells microplate (Tissue Colture Plate 96-wells Flat Bottomwith Lid, Sarstedt, Numbrecht, Germany); microcultures were set in a final volume of 200 µL/well of CCM medium and added with extracts at the desired concentration, or with a DMSO equivalent volume (control). Plates were incubated in the dark under stationary conditions for 6 days at 25 °C. Aflatoxin accumulation was monitored by fluorescence emission determination performed directly from the bottom of wells of the culture plate with a microplate reader (TECAN SpectraFluor Plus, Mannedorf, Switzerland. λ_ex_ = 360 nm; λ_em_ = 465 nm; manual gain = 83; lag time = 0 µs; number of flashes = 3; and integration time = 200 µs). Determination of fluorescence emission/shielding of extracts was tested before proceeding with the assay, in order to exclude any possible interference with the direct fluorescence-based detection of AFs in the CCM culture medium; emission and absorption of extracts at the final concentration of 50, 100, and 500 μg/mL were then assessed at λ_ex_ = 360 nm (aflatoxins specific excitation wavelength) and λ_em_ = 465 nm (aflatoxins specific emission wavelength). The aflatoxin inhibition rate was expressed as a percentage with respect to the control. Treatments were performed in quadruplicate, while plates were settled in triplicate.

### 2.5. Statistical Analysis

The “Past 3.x” software was used to analyse statistical differences between samples (https://past.en.lo4d.com/windows (accessed on 1 October 2021)). Analysis of variance was performed by the Levene test, followed by Tukey’s test [32]. Differences were considered significant at *p* < 0.005.

## 3. Results

### 3.1. Seed Germination In Vitro and Hydroponic Seedlings Cultivation for Extracts Production

**Figure 1:**
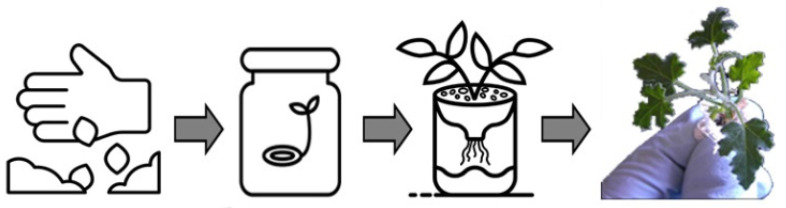
Flowchart of *C. colocynthis* plantlet cultivation. Manually decoated seeds, previously sorted for viability, were soaked and led to germination in sterilized, wetted cotton wool, then transferred into open-top boxes for liquid cultivation until the stage of 6–7 leaves. Credit for icons: noun-plant-seeds-5083968 by Black, noun-plants-1731784 by BGBOXXX Design and noun-hydroponic-1890773 by Wahyu Hadi from “The Noun Project” (https://thenounproject.com (accessed on 17 July 2022)).

Amongst the various procedures applied to hard crust seeds in order to break dormancy and induce germination, decoating was the only one effective in achieving a 100% germination rate in *C. colocynthis* [33,34], as long as chemical, thermal, and sand scratching treatments don’t exceed 60% [35]. However, these studies, that provide methods for the establishment of axenic *C. colocynthis* cultures for micropropagation and cultivation purposes, include the use of plant hormones (auxins and cytokinins alone or in combination) and culture media (MS or Hoagland’s solution). In this study, the use of wetted cotton wool to germinate decoated seeds resulted in a 100% germination response without the support of any synthetic culture medium or chemical additives: shoots initiated from apices and primary roots with significant branches that were developed after 7 days allowed for the effective transfer of plantlets to the open-top boxes for the liquid cultivation system, until the stage of 6–7 leaves (Figure 1).

Extracts were obtained from 7 g lyophilized plantlets and 2 g seed coats (tegument): the extraction yield obtained was settled at 1.057 g for ethanol-extracted plantlets (PL-EtOH) and 0.871 g for chloroform-extracted (PL-CHL), while with ethanol-extracted teguments (Teg-EtOH) the extraction yield obtained was 1.100 g.

### 3.2. Antioxidative and Phytochemical Characterization of C. colocynthis Extracts

**Figure 2:**
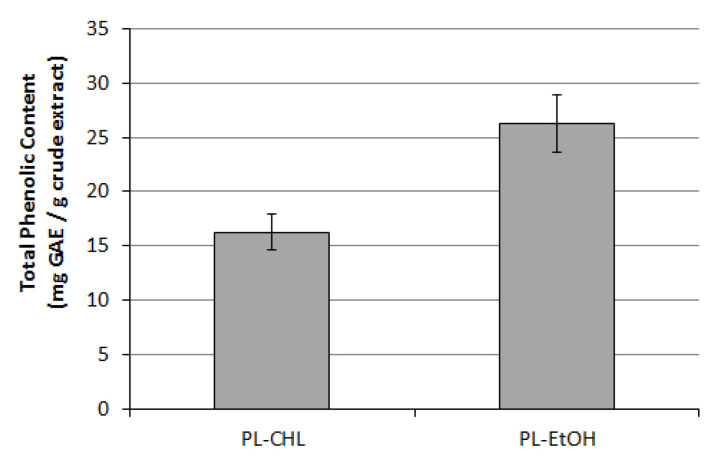
Total Phenolic Content of chloroform (CHL) and ethanol (EtOH) extracts of *C. colocynthis* plantlets. Values are expressed as mg of gallic acid equivalents (GAE)/g of extract ± S.D. (vertical bars; *p* < 0.005).

As the two organic extracts from *C. colocynthis* plantlets were compared, differences higher in total phenolics content than in scavenging activity were found: the ethanol extract (PL-EtOH) showed to possess 26.3 GAE with respect to 16.2 recorded for chloroform extract (PL-CHL), accordingly to the different affinity of solvents with phenolic compounds contained into plantlet tissues (Figure 2).

**Figure 3:**
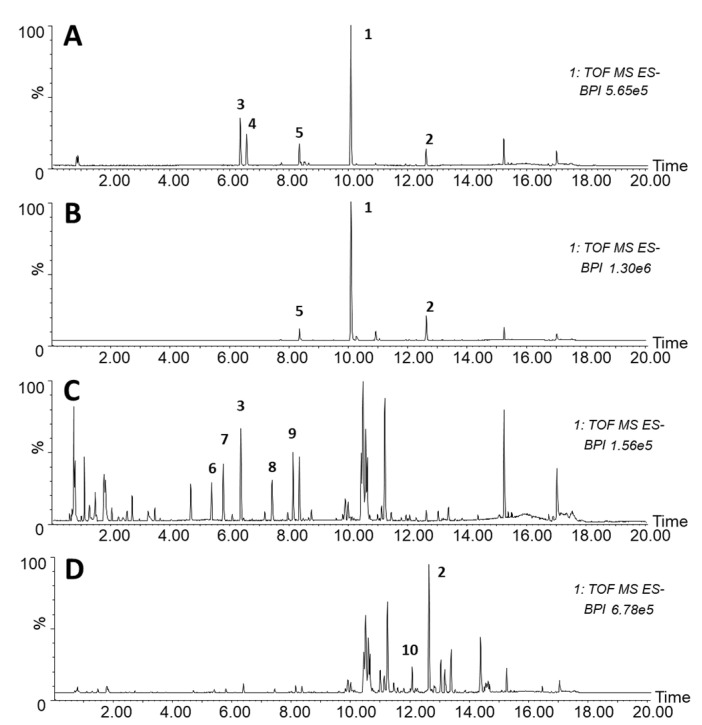
Negative LC-MS base peak chromatograms of *C. colocynthis* extracts. (**A**)**:** ethanol-extracted seed tegument (Teg-EtOH); (**B**)**:** adult leaves extracted in chloroform (Leaf-CHL); (**C**)**:** ethanol-extracted plantlets from in vitro cultures (PL-EtOH); (**D**)**:** chloroform-extracted plantlets from in vitro cultures (PL-CHL). Numbers indicate the following metabolites: (1) Cucurbitacin E 2-O-glucoside; (2) Cucurbitacin E; (3) Apigenin-6-C-glucoside; (4) Isoorientin 3′-O-methyl ether; (5) Cucurbitacin I 2-O-glucoside; (6) Benzyl alcohol hexosyl pentoside; (7) Luteolin-C-hexoside; (8) Methyl apigenin-C-hexoside derivative; (9) Hydroxy-methyl-methoxyflavone-C-hexoside derivative; (10) Cucurbitacin B formic acid adduct.

When subjected to chromatographic analysis, extracts showed different profiles depending both on the starting material and the type of solvent used in the extraction protocol. As shown in Figure 3, chromatograms of seeds and adult leaves extracted respectively in ethanol and chloroform showed similar profiles (Figure 3A,B). In particular, the most abundant peak observed in leaves extracted with chloroform was identified as Cucurbitacin E 2-O-glucoside (*m*/*z*: 763.3532), followed by the formic acid adduct of Cucurbitacin E (*m*/*z*: 601.3008). Likewise, a great abundance of these two molecules was also observed in the ethanol extracts of seed samples, with the Apigenin-6-C-glucoside (*m*/*z*: 431.0971), Isoorientin 3′-O-methyl ether (*m*/*z*: 461.1072), and Cucurbitacin I 2-O-glucoside (*m*/*z*: 721.3426) especially abundant as well. The LC-MS chromatograms of in vitro cultivated seedlings’ ethanol and chloroform extracts revealed several signals that were putatively identified as Benzyl alcohol hexosyl pentoside (*m*/*z*: 401.1442), Luteolin-C-hexoside (*m*/*z*: 447.0918), Apigenin-6-C-glucoside, Methyl apigenin-C-hexoside derivative (*m*/*z*: 553.1340), Hydroxy-methyl-methoxyflavone-C-hexoside derivative (*m*/*z*: 537.1391), Cucurbitacin B formic acid adduct (*m*/*z*: 603.3158), and Cucurbitacin E.

**Figure 4:**
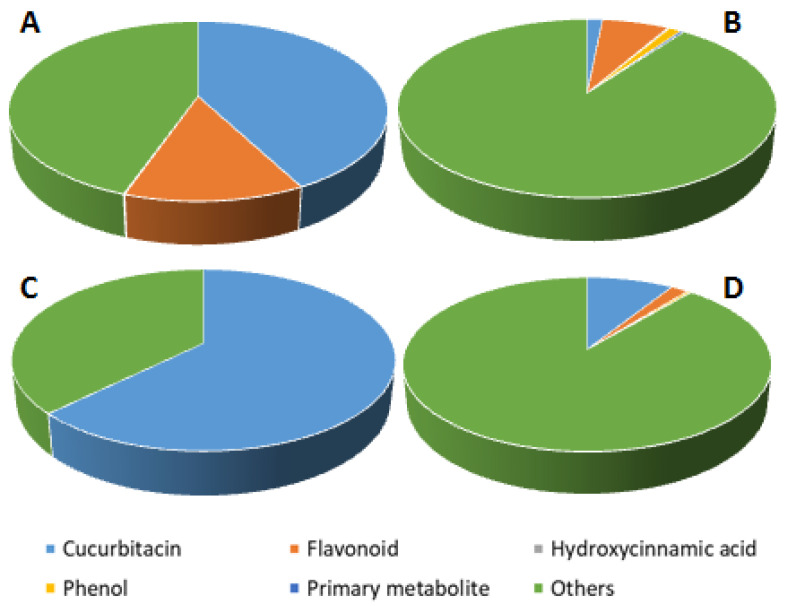
Main metabolite classes detected in *C. colocynthis* extracts. Cake graphs represent the percentages with respect to the total LC-MS signal of the different metabolite classes in: ethanol-extracted seed tegument (Teg-EtOH, (**A**), ethanol-extracted plantlets from in vitro cultures (PL-EtOH, (**B**); adult leaves extracted in chloroform (Leaf-CHL, (**C**)); chloroform-extracted plantlets from in vitro cultures (PL-CHL, (**D**)).

A total of 39 metabolites were putatively identified and for the most part, belonged to the class of cucurbitacins and flavonoids (Supplementary Materials File S1). In particular, the cucurbitacins were particularly abundant in tegument and in adult leaves of wild plants (Figure 4A,B), representing around 42% and 63% of total LC-MS signal, respectively. On the other hand, flavonoids were mainly detected in tegument (13% of total LC-MS signal). The in vitro cultivated seedlings showed a higher presence of flavonoids (7% of total LC-MS signal) in the ethanol extracts (Figure 4C) and a superior accumulation of cucurbitacins in the chloroform extracts (9% of total LC-MS signal; Figure 4D).

### 3.3. Antifungal and Anti-Aflatoxigenic Activity of Extracts

Anticandidal activity of PL-EtOH and PL-CHL is reported as inhibition halo diameter (mm; Table 1). Specie-specific differences were found amongst *Candida* spp.: *C. kreusei* and *C. glabrata* resulted in less susceptibility to both extracts if the effect of extracts is compared with AmpB (14.5 and 12.5 vs. 18.3 mm and 14.7 and 15.0 vs. 21.7 mm, respectively). On the contrary, *C. albicans* and *C. parapsilosis* showed to be differently affected by the two extracts on the basis of solvent used: in fact, while the first was more inhibited by PL-EtOH than PL-CHL (29.0 and 19.0 mm vs. 20.0), the latter displayed an opposite behavior (12.3 and 19.7 mm vs. 15.2).

When it came to assaying the extracts’ bioactivity against the aflatoxigenic phytopathogenic fungus *A. flavus*, two additional organic extracts were added to the analysis: plantlets extracts were tested on *A. flavus* growth and aflatoxin accumulation at 500 µg/mL concentration and compared to seed tegument (Teg-EtOH) and adult leaf (Leaf-CHL) extracts at the same concentration. Leaf-CHL, obtained from mature leaves recovered from wild plants, was previously characterized [12] for its effect on the fungal species and served as an internal reference.

As reported in Table 2, none of them resulted in a significant reduction of mycelium radial growth, suggesting that the composition of the extracts did not provide any appreciable containment potential against the fungus in the tested conditions.

Possible effects on the aflatoxin accumulation by *A. flavus* were assayed in a multiwell, fluorescence-based system, that is particularly suitable for this purpose [13].

**Figure 5:**
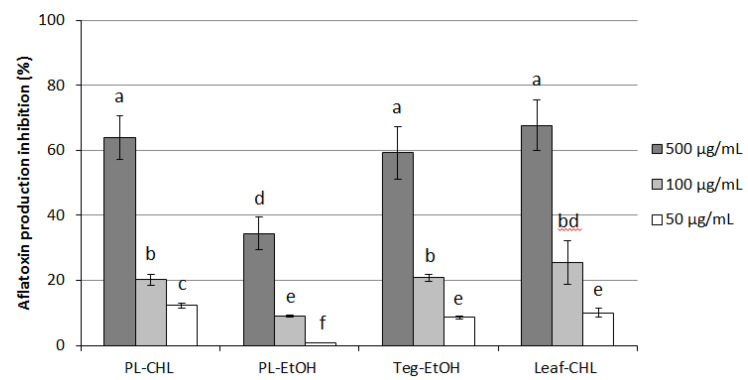
Anti-aflatoxigenic activity of chloroform (PL-CHL) and ethanol (PL-EtOH) extracts of *C. colocynthis* plantlets, mature leaf (Leaf-CHL), and seed coat (Teg-EtOH) at increasing concentrations. Values are means of four replicates and are expressed as inhibition percentage with respect to control ± S.D. Different superscript letters indicate statistically significant differences (*p* < 0.005).

In this case, plantlets, tegument, and leaf extracts administrated at increasing concentrations (50 to 500 µg/mL) were found to exert a dose-dependent inhibitory effect against the toxin, as reported in Figure 5. The highest dose proved to be the most effective for almost all extracts (60 to 70% inhibition), except for PL-EtOH, which became less active (below 40% inhibition) and, as a general observation, the less potent when compared with other extracts at any concentration tested. A similar correlation between solvents used during the extraction procedure and aflatoxin inhibition rate was not really surprising, is already observed in studies previously conducted on *C. colocynthis* extracts [12].

## 4. Discussion

In the current scenario of microbial resistance increase, which is leading to an augmented vulnerability of humans in terms of drug exploitation for the treatment of clinically relevant infections, it is clear how, alongside continuous mining of bioactive plant metabolites, of paramount importance is the development of alternative methods for the cultivation of those medicinal plant species; in fact, due to the various aspects affecting the plants growing wild yield (such as adverse environmental factors, abiotic, and biotic diseases, and differences in developmental stages), cultivation systems allowed to obtain pharmacologically interesting bioactives in an accessible and affordable manner, and, in the case of overharvested species vulnerable to extinction, without undermining natural population levels, are highly desirable. This may also apply to *C. colocynthis*, whose overexploitation for traditional medicine purposes coupled with the rapid urbanization of its environment is rapidly leading to a drastic decrease in local populations. Despite representing an alternative for the production of pharmacological metabolites through the rapid propagation of a large number of uniform plants under controlled conditions, the in vitro culture technology referred to *C. colocynthis* species is poorly applied to date, and not investigated at all for its potential bioactives production, mainly due to the difficulties that govern seed germination in such species. Micropropagation of axenic plants has been achieved after the sterilization of mature tissues (generally nodal segments with axillary bud) collected in the plant’s natural environment, and plantlets were maintained in MS basal medium supplemented with growth promoters before being transplanted in soil [33,36,37]; more recently, conditions for seed germination, callogenesis, organogenesis, and acclimatization of *C. colocynthis* were studied in order to optimize the growth conditions for the micropropagation of this medicinally significant plant [34]. Unlike what reported so far [33,36,38], the obtaining of plantlets with a well-developed root apparatus and normal shoot elongation was here achieved without supplementing the MS medium with phytohormones, such as cytokinins or auxins, suggesting that a less “chemicals”-consuming management of *C. colocynthis* cultures could be successfully applied.

Some of the pharmacologically interesting bioactives found in *C. colocynthis* have been widely proposed to be produced in vitro by tissue cultures—such as undifferentiated calli or regenerated explants—from other species: for example, cucurbitacins were obtained in *Trichosanthes cucumerina* L. var. *cucumerina* callus cultures, stimulated with 2,4-dichlorophenoxy acetic acid in combination with kinetin [21], as well as in *Ecballium elaterium* similar cultures [39]. On the other hand, biochemical profiling was reported for tissues from both in vivo (leaf, stem, fruit, and root) and in vitro (callus and differentiating callus) *C. colocynthis* cultures, revealing significant differences in the amount/distribution of total soluble and reducing sugars, starch, and α-amylase activity [36], but the phytochemical determination of plant extracts from in vitro germinated and hydroponically grown plantlets, along with their antioxidant properties and anti-microbial activity, hadn’t been provided yet.

The secondary metabolome of *C. colocynthis* revealed metabolites falling into two main classes: cucurbitacins and flavonoids. These two groups include compounds that may exert beneficial activities on human health. Flavonoids accumulated in leaf and seed tegument of *C. colocynthis* are mainly apigenin and luteolin derivatives, as also previously reported [17,18]. These aglycones, belonging to the flavone subclass, are known to play several positive activities on human well-being, as described in recent reviews [40,41]; studies performed on humans revealed that apigenin in particular seems to exert in vivo ameliorative effects in cognitive performances, reduces the anxiety disorder symptoms, and shows modest benefits against insomnia [42]. On the other hand, in vivo studies conducted on animals highlighted the beneficial effects of luteolin on glycolipid metabolic dysfunction [41].

Cucurbitacins and cucurbitacin glycosides display several pharmacological properties ranging from anti-ulcer and anti-inflammatory activities to anti-diarrheal and anti-cancer ones. Additionally, cucurbitacins were found to be active against soil-borne pathogens, various insects, and fungi [43]. The biological activities exerted by cucurbitacins are probably related to their chemical nature [27]: in fact, their hydrophobic features might positively drive the absorption of these compounds through biological membranes and into cells. Our results highlighted how the use of chloroform as solvent results in higher extraction yields for cucurbitacins, thus reflecting the highest hydrophobic nature of cucurbitacins [44]. Ethanol has a higher polarity index and better-extracted flavonoids, which is in line with the statement that certain phytochemicals can be efficiently dissolved in solvents sharing similar polarity indexes [45]. Cucurbitacins have often been reported more biologically active when not glycosylated [27]; however, if experiments conducted in vitro on various cancer cell lines—including colon, breast, lung, and brain—support the effectiveness of cucurbitacins B, D, E, and I against these kinds of tumors [46], some glycosylated forms proved to be active as well, since cucurbitacin glucosides (B and E) showed anti-proliferative effects on human breast cancer cells [47], and recent studies suggested that treatment with Cucurbitacin E-glucoside can also inhibit testosterone-induced benign prostatic hyperplasia in mice [48]. According to Maja et al. [43], the amount of Cucurbitacin E-glucoside in *C. colocynthis* leaves was higher than in the other Cucurbitaceae species, reaching values of 1368.4 µg/g FW: here, we observed that in vitro sprouted seedlings retained the ability to accumulate cucurbitacins, especially cucurbitacin E 2-O-glucoside and its aglycone, suggesting a possible future exploitation of this plant cultivation system for the production and recovery of these peculiar metabolites.

Results obtained in biological assays confirmed an anticandidal activity for both PL-CHL and PL-EtOH, although to different extents; in fact, it has already been reported that *Candida* spp. susceptibility to *C. colocynthis* extracts greatly relies on the species and, within the same species, on the extraction solvent [10]. On the other side, if antifungal properties of both botanicals and pure compounds isolated from *C. colocynthis* have been variously described against some phytopathogens (for example, a mixture of spinasterol and 22,23-dihydrospinasterol obtained from the leaves [49], our observations didn’t provide any significant cue with respect to the aflatoxigenic species selected in this study. However, findings showing the absence of any antifungal or fungistatic potential of extracts on the *A. flavus* mycelium long-term growth weren’t completely unexpected: in fact, various studies have reported that several compounds, both synthetic and natural, are effective in lowering AF production without apparently interfering with the fungal hyphae elongation or mycelium development [13,50,51,52,53]; this phenomenon has been ascribed to the induction of physiological responses that possibly affect hyphal branching but not apex elongation, and thus result in unaltered colony marginal expansion—which is necessary for efficient colonization and utilization of the substrate. However, the lack of a manifest antifungal effect clearly doesn’t exclude the possibility of an extract’s interaction with other aspects of fungal development. As demonstrated here, the anti-aflatoxigenic activity of *C. colocynthis* chloroform extracts from in vitro sprouted plantlets was perfectly comparable with that induced by mature leaf extract, supporting the assumption that our cultivation system can be suitable for obtaining organs and/or tissues as rich in bioactive compounds as naturally grown plants. The possibility to exploit plantlets briefly cultivated under controlled conditions rather than more developed organs (such as flowers or seeds [11,13,54] to control the detrimental effects of phytopathogenic fungi on crops and food commodities, offers undeniable advantages in terms of material availability and time-consumption. In turn, the observation that tegument organic extracts can also show an interesting anti-mycotoxigenic potential is particularly remarkable: existing works on seed biological activities focused on whole-seed extracts properties since the manual removal of the external layer is generally too laborious and considered useless [13,55,56].

## 5. Conclusions

Our investigation demonstrated that seedlings sprouted in vitro without the use of culture media and plant hormones are able to accumulate cucurbitacins and in particular the two forms of cucurbitacin E glucoside and the aglycone as well as other cucurbitacins. This finding suggests that this cultivation system might be a useful method to induce accumulation of this peculiar class of metabolites that can be efficiently extracted by using chloroform, or, from a future perspective, with a more environmentally friendly method, for example by using supercritical CO_2_ extraction that is particularly adapted for non-polar metabolites. Additionally, it has been proved that *C. colocynthis* seed coats—possibly deriving from industrial processes—could be profitably recycled for the production of anti-aflatoxigenic formulations. 

## Figures and Tables

**Table 1:** Inhibition halo determined by chloroform and ethanol extracts of *C. colocynthis* plantlets (PL-CHL and PL-EtOH respectively) on *Candida* cultures. Values are expressed, in mm, by means of three replicates ± S.D. Amphotericyn B (AmpB) was used as the control for anticandidal activity. Different superscript letters indicate statistically significant differences (*p* < 0.005).

*Candida* spp. Strain	PL-CHL	PL-EtOH	Amphotericyn B
*C. krusei* ATCC 6258	14.5 ± 0.5 ^b^	12.5 ± 0.6 ^c^	18.3 ± 1.7 ^a^
*C. albicans* ATCC 90028	19.0 ± 1.0 ^a^	29.0 ± 1.0 ^d^	20.0 ± 0.5 ^a^
*C. parapsilosis* ATCC 22019	19.7 ± 1.5 ^a^	12.3 ± 0.6 ^c^	15.2 ± 1.3 ^b^
*C. glabrata* ATCC 90030	14.7 ± 0.6 ^b^	15.0 ± 1.0 ^b^	21.7 ± 1.0 ^a^

**Table 2:** Radial growth of *A. flavus* colonies exposed to *C. colocynthis* plantlets chloroform (PL-CHL) and ethanol (PL-EtOH) extracts, seed tegument ethanol extract (Teg-EtOH) and leaf chloroform extract (Leaf-CHL) at 500 µg/mL concentration. The radial increment is expressed as the mean of daily radial increase of colonies radius (mm/d) ± S.D. The same superscript letters indicate the absence of statistically significant differences (*p* < 0.005).

PL-CHL	PL-EtOH	Teg-EtOH	Leaf-CHL	CNT
50.3 ± 2.8 ^a^	49.4 ± 1.6 ^a^	47.0 ± 1.7 ^a^	46.5 ± 1.5 ^a^	44.9 ± 3.5 ^a^

## Supplementary Materials

The following supporting information can be downloaded at: https://www.mdpi.com/article/10.3390/plants11202711/s1, Supplementary File S1: list of the 39 metabolites identified.
